# Leadership, regulatory approaches and policy to re-orientate health services towards health promotion

**DOI:** 10.1093/heapro/daae050

**Published:** 2024-05-29

**Authors:** Chun Han, Clare Lin, Anil Raichur, Martin Hall, Tan Minh Nguyen

**Affiliations:** Dental Health Services Victoria, Level 1, 720 Swanston Street, Carlton, Victoria, 3053, Australia; Dental Health Services Victoria, Level 1, 720 Swanston Street, Carlton, Victoria, 3053, Australia; Melbourne Dental School, University of Melbourne, Level 1, 720 Swanston Street, Carlton, Victoria, 3053, Australia; Victorian Department of Health, 50 Lonsdale Street, Melbourne, Victoria, 3000, Australia; Dental Health Services Victoria, Level 1, 720 Swanston Street, Carlton, Victoria, 3053, Australia; Melbourne School of Population & Global Health, Faculty of Medicine, Dentistry and Health Sciences, University of Melbourne, 207 Bouverie Street, Carlton, Victoria, 3053, Australia; Dental Health Services Victoria, Level 1, 720 Swanston Street, Carlton, Victoria, 3053, Australia; Institute for Health Transformation, Deakin Health Economics, Deakin University, 221 Burwood Highway, Burwood, Victoria, 3125, Australia; Health Economics Group, School of Public Health and Preventive Medicine, Faculty of Medicine, Nursing and Health Sciences. Monash University, Level 4, 553 St Kilda Road, Melbourne, Victoria, 3004, Australia

**Keywords:** health policy, regulation, leadership, public health practice, health promotion

## Abstract

Globally, oral conditions remain the most prevalent of all non-communicable diseases. Among the broad range of target goals and recommendations for action by the World Health Organization’s Global Oral Health Strategy, we call out three specific actions that provide an enabling environment to improve population oral health including: (i) enabling population oral health reform through leadership, (ii) enabling innovative oral health workforce models, (iii) enabling universal health coverage that includes oral health. The aim of the article is to outline how leadership, regulatory approaches and policy in Australia can strengthen health promotion practice and can inform global efforts to tackle the complex wicked problems associated with population oral health. Examples in Australia show that effective leadership, regulatory approaches and well-designed policies can address the growing burden of non-communicable diseases, and are made possible through public health advocacy, collaboration and research.

**Contribution to Health Promotion**

- The COVID-19 pandemic raises important considerations for oral healthcare as being an essential health service.
- Health promotion can be enhanced by having an enabling environment through leadership, regulatory approaches and policy.
- Mutli-layered approaches to health promotion are critical to embed and prioritize oral health as a public health issue.

## INTRODUCTION

For the last three decades, the combined global prevalence of non-communicable diseases (NCDs) for oral conditions, such as dental caries (tooth decay), periodontal (gum) disease and severe tooth loss, has remained the highest of all NCDs, at around 45% (World Health Organization, 2022a). This is equivalent to 3.5 billion cases of oral diseases and related conditions, most of which are largely preventable (GBD 2017 Oral Disorders Collaborators *et al*., 2020) by addressing modifiable risk factors and the social determinants of health.

Good oral health supports eating, speaking, mental and social well-being, and the ability to socialize and work without pain, discomfort and embarrassment (World Health Organization, 2022a). Despite the profound effects of poor oral health on general health and wellbeing, governments have largely failed to prioritize population oral health, with the administration and financing of oral healthcare remaining siloed rather than integrated into healthcare systems (Benzian *et al*., 2022; Nguyen *et al*., 2023a).

The 1986 Ottawa Charter for Health Promotion (World Health Organization, 2024a) serves as a guiding framework for health promotion framework worldwide. The charter identified five key action areas for health promotion including building healthy public policy and reorienting health services, of which this article focuses on. The 2005 Bangkok Charter for Health Promotion in a Globalized World in Bangkok (World Health Organization, 2024b) reinforces efforts should be directed to broad key areas including regulatory and legislation levers and building capacity, which includes health professionals. Our article links the importance of strengthening health promotion practices at the health service level including oral health.

The World Health Organization (WHO) led a landmark global policy direction outlined in the Global Oral Health Strategy (World Health Organization, 2022a), and subsequently, the development of the Global Oral Health Action Plan 2023–30 (World Health Organization, 2022b). Amongst a broad range of target goals and recommendations for action for Member States, we call out three specific actions that provide an enabling environment to improve population oral health through leadership, regulatory approaches and policy (World Health Organization, 2022b). We believe the following actions stand out as the most effective, significant and feasible strategies, essential to drive positive change and re-orient health services towards a stronger emphasis on health promotion that includes oral health:

- Action 2—Strengthen national oral health leadership;
- Action 45—Foster innovative oral health workforce models;
- Action 63—Work towards achieving Universal Health Coverage for oral health.

The aim of this article is to outline how leadership, regulatory approaches and policy in Australia can strengthen health promotion practice and can inform global efforts to tackle the complex wicked problems associated with population oral health (Baker, 2019).

## ENABLING POPULATION ORAL HEALTH REFORM THROUGH LEADERSHIP

Effective leadership is necessary for healthcare organizations (Kumar and Khiljee, 2016), including the oral health sector (Kalenderian *et al*., 2013; Williams *et al*., 2019). The WHO’s Oral Health Action Plan 2023–30 set a target that ‘By 2030, 80% of countries have an operational national oral health policy, strategy or action plan and dedicated staff for oral health at the Ministry of Health or other national governmental health agency’, and to appoint an officer within the oral health unit (World Health Organization, 2022b). In the Australian context, the absence of an Australian Chief Oral Health Officer reflects a lack of prioritization for oral health and has hampered nationally coordinated approaches to improve population oral health (Nguyen *et al*., 2023b). As an example, specific reference to the prevention of oral diseases is largely absent from Australia’s first National Preventive Health Strategy 2021–30 (Australian Government Department of Health, 2021).

Health is a complex adaptive system (Bircher and Kuruvilla, 2014), and therefore, a systems approach for oral disease prevention is most likely to have the greatest population health impact at the lowest cost. However, a citation analysis paper found that oral health research does not often seem to be translated to Australian oral health and chronic disease policy (Ingram *et al*., 2021). Population interventions for oral disease prevention, such as community water fluoridation, are unequivocally cost-effective, when compared to individual-based interventions (Nguyen *et al*., 2023c). However, translating effective population health interventions into the real world and at scale can be challenging due to a lack of awareness and translation of evidence, lack of leadership and advocacy, budgetary pressures, competing priorities and lack of supportive health policy.

In the absence of national leadership in Australia, equivalent local state and territory oral health roles, together with leadership from other key stakeholders, have had a positive impact on local population oral health initiatives. For example, in Victoria, the explicit inclusion of community water fluoridation in a local government Municipal Public Health and Wellbeing Plan (along with advocacy efforts) led to a funding commitment by the state government to install a fluoridation plant for the community (Dickson‐Swift and Crocombe, 2022). Previous research identified that low prioritization or the absence of oral health from Municipal Public Health and Wellbeing Plans has perpetuated the invisibility of oral health as a public health issue (Nguyen *et al*., 2022).

Value-based health care is another opportunity for oral health reform requiring strong leadership. It focuses on achieving the health outcomes that matter most to consumers relative to the costs to deliver these outcomes (Gray, 2006). Globally, the adoption of value-based health care for the oral health context has been limited but shows promise and could support progress to achieve universal health coverage that includes oral health (Nguyen *et al*., 2023a). Successful implementation of value-based health care requires strong leadership, significant organizational commitment and effort and priority alignment (Renting *et al*., 2022). From a population oral health perspective, effective health promotion is pivotal but remains absent from the value-based health care literature. There is a need to strengthen health promotion at community and individual levels.

## ENABLING INNOVATIVE ORAL HEALTH WORKFORCE MODELS

Building healthy public policy is the overarching action area of the Ottawa Charter and intervention can occur through legislation and regulation (Rogers, 2023). Dental practitioners have a critical role in both service delivery and health promotion to improve the population oral health. In addition, health practitioner regulation plays a critical role in protecting patients (Mahat *et al*., 2023) and promoting high-quality health care. However, policies related to health practitioner regulation are typically influenced, if not driven by the health professions, with limited input from consumers or consideration of the overall population’s health needs. Australia’s approach to health practitioner regulation through the National Regulation and Accreditation Scheme was a landmark achievement from an international perspective. Formalized in 2010, it is one of few health practitioner regulation agencies that include consumer members on the health practitioner profession boards (Mahat *et al*., 2023). However, health practitioner regulation in Australia has been criticized for being slow, and unable to keep up with the dynamic health needs of consumers (Wardle *et al*., 2016).

For the dental profession, we do see evidence of slow progress, but also significant opportunities where regulatory approaches can make impactful changes to the healthcare system to address the changing needs of the population. The WHO Global Oral Health Action Plan 2023–30 recommends the need to foster innovative oral health workforce models and encourage skill mix. The plan also proposes the action to review and update national legislative and regulatory policies for licensing, accreditation and scopes of practice to support flexible workforce models (World Health Organization, 2022b). In 2011, the Australian government commissioned a report on the Scope of Practice Review on oral health practitioners (dental therapists, dental hygienists and oral health therapists). It recommended that the Dental Board of Australia remove the bar to independent practice within 5 years for dental therapists, dental hygienists and oral health therapists (Health Workforce Australia, 2011). Previous modelling has shown that the potential cost-savings from increased utilization of an oral health therapists workforce could be reinvested to deliver more oral healthcare and reduce the public adult dental waiting lists (Nguyen *et al*., 2019).

In Australia, the oral health therapist workforce has gained widespread recognition for delivering high-quality and cost-effective dental services within their defined scope of practice. Oral health therapists have combined competencies in dental hygiene and dental therapy, which includes oral health promotion. They provide routine oral healthcare including dental examinations, preventive procedures, placement of restorations and non-surgical periodontal treatments, alongside dentists and dental prosthetists as part of the dental team (Dental Board of Australia, 2020). Prior to 2020, prohibited access to direct billing rights to government dental programs and private health insurances rebates has meant that dental hygienists, dental therapists and oral health therapists (Health Workforce Australia, 2011) were usually restricted to providing oral healthcare as dictated by their employer’s business practice arrangements.

The regulatory bar on independent practice was eventually rescinded under the leadership of the Dental Board of Australia in 2020 (Dental Board of Australia, 2020). Direct billing rights for dental hygienists, dental therapists and oral health therapists consequently occurred in 2022 under the Australian government’s Child Dental Benefits Scheme (Australian Government Department of Health, 2022), a means-tested dental program. To date, Australian government dental schemes have only recognized dental practitioners in providing oral healthcare, and not included the preventive oral health services that could be performed by relevant non-dental practitioners in primary care. This would provide an important mechanism for integrating oral health into primary care to support the population oral health.

A significant part of Australia’s healthcare system relies on internationally trained health professionals, including dentists. Dentists with an overseas dental qualification make up at least a quarter of the overall dental workforce in Australia (Balasubramanian *et al*., 2021) and have contributed to the public oral health workforce, particularly in rural and remote areas. In 2022, the National Cabinet announced an independently led review of Australia’s regulatory settings, covering health practitioner registrations and skill and overseas qualification recognition. The interim report suggests that immediate actions are needed from governments and regulators to accelerate the Australian registration process, including eliminating duplicative steps and fast-tracking more applicants from countries with similar regulatory systems (Kruk, 2023).

These reforms are crucial for medicine and nursing professions, but its relevance for the dental profession is less obvious because most dental practitioners work in private practice. The oral health therapist workforce is also underdeveloped internationally both in terms of professional identity and workforce size. Innovative ways of leveraging overseas-trained dentists in Australia should be explored, particularly for candidates who face difficult challenges to successfully pass the Australian Dental Council’s examination process, which is required for general registration (Balasubramanian *et al*., 2014). In addition, the ethical recruitment of skilled health professionals must consider (Cooper *et al*., 2020) and adhere to the WHO Global Code of Practice on the International Recruitment of Health Personnel (World Health Organization, 2010).

For example, one regulatory policy option could be to consider providing a pathway for general registration, initially with conditions. This would mean that overseas-trained dentists could incrementally be accredited to work with the limited scope of practice with core dental practitioner competencies including oral health promotion, participate in the health workforce more readily and enable skills development and confidence to expand to the broadest scope of practice as a dentist, if desired. This approach can achieve one of the objectives of the National Regulation and Accreditation Scheme ‘to enable the continuous development of a flexible, responsive and sustainable Australian health workforce and to enable innovation in the education of, and service delivery by, health practitioners’ (Standing Council on Health, 2014).

## ENABLING UNIVERSAL HEALTH COVERAGE THAT INCLUDES ORAL HEALTH

The social norm that oral healthcare is primarily the responsibility of the dental profession needs to change. As Marmot and Allen argue, poor oral health is a public health equity issue (Marmot and Allen, 2014). Changing the paternalistic narrative that dentists are the *only* health professionals involved in providing oral healthcare will be pivotal to achieving universal health coverage that includes oral health. For example, the peak international organization for oral health, the FDI World Dental Federal, only accepts national dental associations representing dentists as the core membership base (FDI World Dental Federation, 2024). In addition, dental hygienists, dental therapists and oral health therapists are referred to as ‘mid-level’ providers in WHO’s Global Oral Health Strategy (World Health Organization, 2022a) and Oral Health Action Plan 2023–30 (World Health Organization, 2022b). It is not surprising that most, if not all, media articles related to dental services or oral health topics would reference *the* dentist and any supporting commentary is typically made by dentists.

Beyond the oral health team, it is our view that oral health should be integrated into mainstream primary care (Nguyen *et al*., 2023a), to end the illogical separation of the mouth from the rest of the body (Vieira and Caramelli, 2009). This would require the medical and nursing profession to take a leadership role by working collaboratively with the dental profession to strengthen oral health within curricula and embed oral health within health promotion practice (Peckham *et al*., 2017). There has been considerable progress in integrating oral health by non-dental professionals (Silk, 2017). For example, the US Preventive Services Task Force recommends that primary care providers apply fluoride varnish to the teeth of all infants and children starting as soon as deciduous (baby) teeth erupt up to age 5 years to prevent dental caries (US Preventive Services Task Force *et al*., 2021).

Fluoride varnish is a high-concentration sodium fluoride paste, which helps to prevent dental caries and is listed on the WHO’s Essential Medicines List (World Health Organization, 2023). When fluoride varnish is applied at least twice a year, it can yield significant dental caries prevention benefits (Marinho *et al*., 2013). In many countries and jurisdictions, fluoride varnish is not available or permitted for use by non-dental practitioners. Leadership and advocacy are needed to drive amendments to drugs and poisons regulations to permit the use of concentrated fluorides by non-dental practitioners, either at a national or jurisdiction level.

It should be noted that approval processes to amend the drugs and poisons regulations on fluoride varnish to expand the scope of practice of non-dental practitioners and enable effective health promotion can be lengthy (Ummer-Christian *et al*., 2024). There can also be resistance as evident in this statement by one of the peak health profession associations: ‘Professional topical application of fluorides must be selectively used on patients who, as a result of an evaluation conducted by a dentist, (or other appropriately trained dental practitioners) are assessed as having an increased risk of tooth decay’ (Australian Dental Association, 2023).

In Australia, fluoride varnish is a Schedule 4 medicine under the Therapeutic Goods Administration and restricted to use by registered medical practitioners but is considered Schedule 3 when used by registered dental practitioners. Various states and territories have revised their drugs and poisons regulations to enable registered non-dental practitioners and unregistered health professionals to use fluoride varnish (Skinner *et al*., 2021). For example, Aboriginal Health Workers and nurses in the Northern Territory and Western Australia are authorized access to apply fluoride varnish to children (Nguyen, 2017). In Victoria, dental assistants with relevant certificate IV qualifications can apply fluoride varnish to children aged 3–17 years from 2018. More recent regulatory amendments extended this to include registered Aboriginal and Torres Strait Islander health practitioners in 2022 (Victorian Department of Health, 2023; Ummer-Christian *et al*., 2024).

Previous research demonstrated that regular application of fluoride varnish is cost-effective for the Australian context (Nguyen *et al*., 2020) and is clinically effective, particularly for higher-risk populations including Aboriginal communities (Slade *et al*., 2011). In 2022, Dental Health Services Victoria made a formal application to the Medicare Benefits Schedule Review Advisory Committee requesting the Committee include the application of fluoride varnish in the Medicare Benefits Schedule to enable registered non-dental practitioners to claim reimbursement benefits for this service. However, the lack of recognition that oral health is a federal government responsibility formed the basis for the application to not progress further. Similarly, the Public Health Association of Australia made an unsuccessful submission to the Therapeutic Goods Administration in 2023 to make a minor amendment to The Poisons Standard. The proposed simple amendment would replace ‘registered dental practitioners’ with ‘registered health practitioners’.

## CONCLUSION

Improving population oral health requires a multifaceted approach involving leadership, regulatory measures and well-designed policies. Key considerations include public health advocacy—advocate the importance of oral health as a crucial component of health, intersectoral collaboration—fostering collaboration between dental practitioners, the health workforce, healthcare providers, educators and policymakers, research and innovation—encourage research into effective prevention, treatments and health promotion strategies.
